# A Decision-Oriented Calibrated Screening Workflow for Tylosin Derivatives: Closed-Loop MIC Validation Against *Staphylococcus aureus* and *Streptococcus agalactiae*

**DOI:** 10.3390/antibiotics15070666

**Published:** 2026-07-08

**Authors:** Huan Liu, Yiming Liu, Na Yu, Miao An, Yaoxin Tang, Jing Qu, Xiubo Li

**Affiliations:** 1 National Feed Drug Reference Laboratories, Feed Research Institute, Chinese Academy of Agricultural Sciences, Beijing 100081, China; 82101231342@caas.cn (H.L.); 821012420002@caas.cn (N.Y.); anqijia@webmail.hzau.edu.cn (M.A.); 821012410486@caas.cn (Y.T.); 821012430496@caas.cn (J.Q.); lixiubo@caas.cn (X.L.); 2 Key Laboratory of Animal Antimicrobial Resistance Surveillance, Ministry of Agriculture and Rural Affairs, Feed Research Institute, Chinese Academy of Agricultural Sciences, Beijing 100081, China; 3 Laboratory of Quality and Safety Risk Assessment for Products on Feed-Origin Risk Factor, Ministry of Agriculture and Rural Affairs, Feed Research Institute, Chinese Academy of Agricultural Sciences, Beijing 100081, China

**Keywords:** tylosin derivatives, antimicrobial screening, calibrated probability, applicability domain, *Staphylococcus aureus*, *Streptococcus agalactiae*

## Abstract

**Background:** Antimicrobial resistance among Gram-positive veterinary pathogens has increased the need for more rational and experimentally grounded strategies to prioritize tylosin derivatives for antibacterial development. This study aimed to develop and externally a calibrated, decision-oriented screening workflow for tylosin-derived antibacterial analog prioritization against *Staphylococcus aureus* and *Streptococcus agalactiae*. **Methods:** Publicly available minimum inhibitory concentration (MIC) data were used to construct organism-specific multilayer perceptron models. Model performance was evaluated using a five-fold out-of-fold framework. Calibrated activity probabilities were then combined with precision-oriented thresholds and similarity-based applicability-domain constraints to define prospective Go/No-Go decisions. To examine external transferability, six synthesized tylosin derivatives (A1–A6) were prospectively predicted and subsequently tested using in vitro MIC assays. **Results:** Internal validation showed stronger and more stable performance for *S. aureus* than for *S. agalactiae*. However, closed-loop external validation revealed distinct organism-specific decision behaviors. For *S. aureus*, the workflow assigned Go decisions to five compounds and included the only experimentally active analog, A6, but also generated several false Go decisions for inactive analogs. For *S. agalactiae*, all six compounds were classified as No-Go under the primary decision rule, whereas MIC testing showed that four analogs were experimentally active, indicating conservative under-selection in a low-data extrapolation setting. **Conclusions:** Calibrated probabilities and applicability-domain analysis can improve the transparency and diagnostic value of antibacterial prioritization, but they do not guarantee robust external activity prediction when training-set coverage, threshold transferability, or chemical-space representation is limited. Overall, this study provides an early-stage, risk-aware closed-loop decision-support framework for tylosin-derived analog prioritization and highlights the need for iterative model updating, probability recalibration, and larger external validation before such workflows are used as high-confidence prospective screening tools in veterinary antibacterial discovery.

## 1. Introduction

Bovine mastitis remains one of the most important diseases in dairy production because it reduces milk yield and quality, increases treatment and culling costs, and imposes persistent animal-health and management burdens on dairy farms [1,2,3]. The disease is still predominantly bacterial in origin, and staphylococci, streptococci, and coliforms continue to dominate the etiological spectrum reported across dairy systems [2,3]. Because mastitis remains one of the major reasons for antimicrobial use in dairy cattle, the continuing rise in antimicrobial resistance further increases the need for more rational pathogen-directed therapeutic and discovery strategies [3].

Among mastitis-associated pathogens, *Staphylococcus aureus* and *Streptococcus agalactiae* remain especially important because both are classically regarded as contagious mastitis agents and can persist within herds through cow-to-cow transmission [1,4]. Large-scale and regional studies continue to identify these organisms among the major mastitis-associated bacteria, although their relative contribution varies across countries, management systems, and clinical contexts [4,5]. Historical herd-level surveys likewise established *S. aureus* and *S. agalactiae* as major contagious mastitis pathogens, and that epidemiological importance has not disappeared despite shifts in broader mastitis ecology [1,6].

*Staphylococcus aureus* is particularly challenging because its epidemiology and pathogenicity are shaped by strain diversity, host adaptation, intracellular persistence, and multiple virulence-associated traits, all of which complicate treatment and herd-level control [7,8]. Meta-analytical and surveillance work also indicates that bovine mastitis-associated *S. aureus* continues to show substantial antimicrobial-resistance burden in important dairy regions, including China [9]. Drug-resistant *S. aureus*, including methicillin-resistant *S. aureus* (MRSA), represents an even more challenging target for antibacterial discovery and resistance-aware treatment. However, public MIC datasets do not consistently provide strain-level resistance phenotypes, which limits resistance-phenotype-specific modeling in the present workflow. Similarly, molecular characterization studies of bovine *S. agalactiae* have continued to detect antimicrobial-resistance traits and emphasize the need for sustained monitoring of this pathogen in mastitis control programs [10]. Antimicrobial susceptibility studies performed on mastitis isolates further support the clinical relevance of resistance-aware treatment decisions for both *S. aureus* and *S. agalactiae* [11].

Macrolides remain important drugs in veterinary medicine, and tylosin has long been used in food-producing animals. At the same time, tylosin exposure has raised persistent concern about antimicrobial-selection pressure and resistance ecology in livestock systems. Experimental work has shown that in-feed tylosin administration can influence macrolide resistance among enterococci recovered from cattle, and that withdrawal effects may not immediately eliminate this signal [12]. Broader modeling work has likewise concluded that tylosin use in beef production can contribute to resistance-selection dynamics that are relevant to stewardship decisions [13]. More recent randomized field data also indicate that continuous in-feed tylosin use can increase macrolide-resistant enterococci and their fecal shedding under feedlot conditions [14]. Against this background, rational optimization of the tylosin scaffold is attractive because it may help identify more potent analogs while making more efficient use of limited synthetic and biological-testing resources.

In practice, however, optimization of tylosin derivatives still faces a major efficiency bottleneck. Traditional workflows often depend on iterative synthesis followed by compound-by-compound MIC testing, with subsequent structural refinement guided largely by empirical judgment. This process is costly and slow, especially when only a small analog series is available for follow-up. More importantly, even within a single parent scaffold, modest structural changes may lead to highly non-linear changes in antibacterial outcome, making intuitive prioritization unreliable. These constraints are particularly relevant in veterinary antibacterial research, where scaffold-focused series are common but experimental throughput is limited [3,8].

Recent progress in molecular machine learning has made data-driven prioritization increasingly attractive for small-molecule discovery. At the same time, the field has also become more cautious about the difference between strong internal benchmarks and truly reliable deployment behavior. For example, uncertainty-aware molecular prediction studies have shown that neural models may be overconfident and that uncertainty quantification is essential for decision-making under limited or noisy data conditions [15,16]. Architecture-search-based uncertainty frameworks have further emphasized that separating epistemic and aleatoric uncertainty can improve the practical trustworthiness of molecular-property models [17]. More recent evaluation studies have also shown that model rankings and apparent robustness can change substantially under different out-of-distribution splitting strategies, reinforcing the need for realistic assessment before deployment [18].

These issues are highly relevant in antibacterial screening, where public MIC data are often heterogeneous with respect to strains, assay conditions, and chemical series. Under such circumstances, predictive scores are most useful when they can be translated into explicit decisions rather than treated as abstract rankings only. This makes probability calibration, uncertainty awareness, and distribution-sensitive evaluation especially important for prospective compound prioritization [15,16,18]. It also suggests that external validation should not be treated merely as a final checkpoint, but as part of an iterative process that reveals where predictive confidence is justified and where extrapolation risk becomes dominant [17].

Accordingly, the aim of the present study was to develop and prospectively evaluate a calibrated, decision-oriented screening workflow for tylosin derivatives against *S. aureus* and *S. agalactiae*. The novelty of this study lies not in the development of a new molecular-learning architecture, but in the integration of calibrated probability prediction, applicability-domain assessment, explicit Go/No-Go decision rules, and closed-loop experimental MIC validation into a transparent antibacterial prioritization framework. In this study, “decision-oriented” refers to the conversion of calibrated activity probabilities and applicability-domain information into explicit Go/No-Go prioritization decisions for experimental follow-up, rather than the generation of activity scores alone. Using publicly available MIC data, we trained organism-specific classification models and evaluated whether their outputs could support prospective prioritization under realistic extrapolation constraints. Rather than treating external validation as a simple success-or-failure endpoint, we used the six-compound tylosin-derivative series to examine the practical utility, decision behavior, and boundary conditions of model-guided antibacterial prioritization in a veterinary antibacterial-discovery setting.

## 2. Results

### 2.1. Closed-Loop Screening Workflow

**Figure 1:**
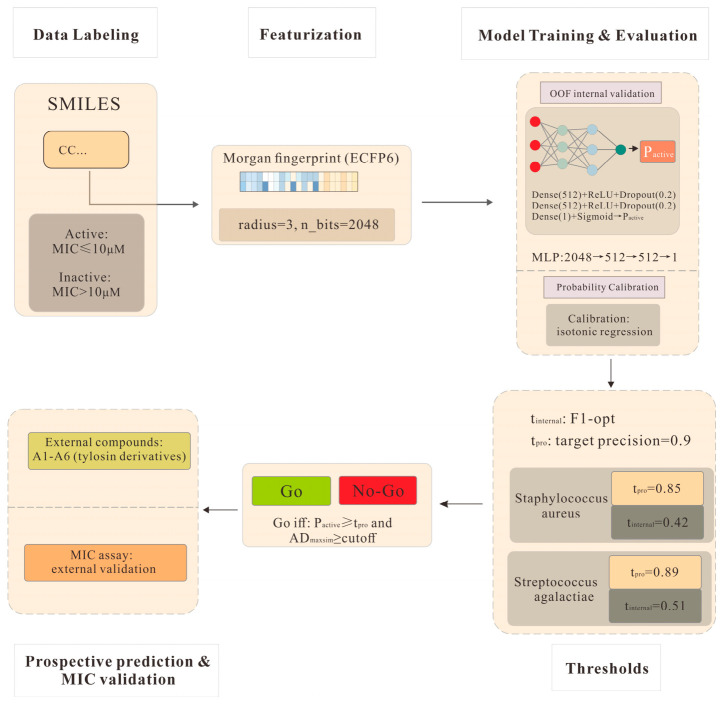
Workflow of the calibrated, decision-oriented screening pipeline. Public MIC data were converted into binary activity labels, molecular structures were encoded as Morgan fingerprints, organism-specific MLP models were trained and calibrated, and calibrated probabilities were combined with applicability-domain constraints to generate Go/No-Go decisions for external MIC validation.

The overall calibrated, decision-oriented screening workflow is shown in Figure 1. Public MIC records were first converted into binary activity labels using the MIC ≤ 10 μM cutoff. Molecular structures were then standardized and encoded as Morgan fingerprints (ECFP6; radius = 3, nBits = 2048), after which organism-specific multilayer perceptron (MLP) classifiers were trained separately for *S. aureus* and *S. agalactiae*. Out-of-fold (OOF) prediction probabilities were then calibrated to generate decision-relevant activity probabilities *p*_active_. To support prospective prioritization, two thresholds were defined: an internal reference threshold *t*_internal_ selected by F1 optimization, and a prospective threshold *t*_pro_ selected to favor high precision. In parallel, a similarity-based applicability-domain metric AD_maxsim_ was used to assess whether external compounds were sufficiently close to the training distribution. Final Go/No-Go decisions were made by combining calibrated probability and AD constraints, and the predictions were subsequently tested by experimental MIC assays on six external tylosin derivatives (A1–A6), thereby forming a closed-loop validation framework.

### 2.2. Internal OOF Validation

**Figure 2:**
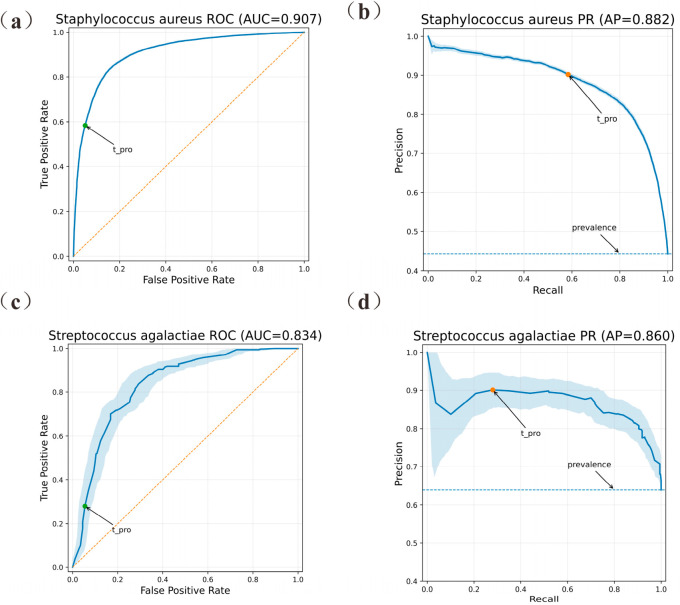
Out-of-fold (OOF) internal validation of calibrated MLP models for *Staphylococcus aureus* and *Streptococcus agalactiae*. (**a**,**c**) ROC curves with AUC. The blue solid lines indicate the ROC curves, and the yellow dashed diagonal lines indicate the random-classifier reference. (**b**,**d**) Precision–recall (PR) curves with average precision (AP); The blue solid lines indicate the PR curves, and the blue dashed horizontal lines indicate the positive prevalence in the training data. The operating point corresponding to *t*_pro_, selected to achieve target precision of 0.90, is highlighted on each curve. Shaded regions (when visible) denote uncertainty bands estimated from resampling/fold-to-fold variability.

In the OOF internal validation, the calibrated MLP models showed good discriminative performance for both Gram-positive organisms, although performance was more stable for *S. aureus* than for *S. agalactiae* (Figure 2; Table 1). For *S. aureus*, which had the larger dataset (*n* = 43,889, positive rate ≈ 0.443), the model achieved a ROC-AUC of 0.907 and an AP of 0.882, indicating strong ranking and enrichment ability despite class imbalance. For *S. agalactiae* (*n* = 559, positive rate ≈ 0.639), the model achieved a ROC-AUC of 0.834 and an AP of 0.860, suggesting that the task remained learnable but was associated with greater uncertainty, consistent with the smaller dataset size and wider variability observed in the validation curves.

To translate calibrated probabilities into actionable screening rules, organism-specific thresholds were determined from the OOF predictions (Table 1). For *S. aureus*, *t*_internal_ and *t*_pro_ were set to 0.42 and 0.85, respectively. For *S. agalactiae*, the corresponding thresholds were 0.51 and 0.89. The operating points associated with *t*_pro_ are highlighted in Figure 2 and reflect the intended use of the workflow in a precision-oriented screening setting. Additional early enrichment analyses further supported the prioritization value of the models, particularly for *S. aureus*, which showed substantial enrichment in the top-ranked fraction of compounds (Table S2A,B).

### 2.3. Prospective External Validation

**Figure 3:**
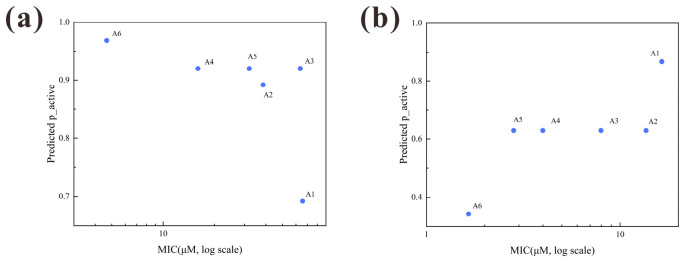
Prospective external validation of tylosin derivatives (A1–A6): relationship between calibrated activity probability (*p*_active_) and experimentally measured MIC values for (**a**) *Staphylococcus aureus* and (**b**) *Streptococcus agalactiae*.

To assess real-world transferability, six synthesized tylosin derivatives (A1–A6) were subjected to prospective prediction and experimental MIC testing. For each compound, the raw probability (*p*_raw_), calibrated activity probability (*p*_active_), applicability-domain similarity (AD_maxsim_), Go/No-Go decision, and experimental MIC value are summarized in Table 2 and visualized in Figure 3. Activity was defined consistently with the training labels as MIC ≤ 10 μM.

#### 2.3.1. External Validation Against *S. aureus*

For *S. aureus*, the external tylosin derivatives generally received high calibrated probabilities. Compounds A2–A6 showed *p*_active_ values ranging from 0.892 to 0.968, all above the prospective threshold (*t*_pro_ = 0.85). In addition, their AD_maxsim_ values ranged from 0.785 to 0.919, all exceeding the primary AD cutoff of 0.30. Under the joint decision rule, these five compounds were therefore classified as Go. In contrast, A1 had an AD_maxsim_ value of 0.86, indicating that it was inside the applicability domain, but its calibrated probability (0.6919) did not reach *t*_pro_, and it was thus classified as No-Go.

Experimental MIC testing revealed substantial variation in antibacterial activity across the same external series, with MIC values ranging from approximately 4.67 to 65.63 μM. Among the six compounds, only A6 met the predefined activity criterion, with an MIC of 4.67 μM, whereas A1–A5 all showed MIC values above 10 μM and were therefore classified as inactive. These results indicate that the workflow retained practical prioritization value in identifying A6 as an active external compound, but also showed optimistic behavior for several structurally related compounds that were assigned Go status despite lacking activity in the experimental assay.

#### 2.3.2. External Validation Against *S. agalactiae*

For *S. agalactiae*, the external behavior was markedly different. The calibrated probabilities were overall more conservative, ranging from 0.3429 to 0.8679, and none of the six compounds reached the predefined prospective threshold of 0.89. At the same time, the AD_maxsim_ values were low for all compounds, ranging from 0.197 to 0.245, which placed all candidates outside the primary applicability domain defined by AD_cutoff_ = 0.30. As a result, all six tylosin derivatives were classified as No-Go under the primary joint decision rule.

However, MIC testing showed that the conservative prediction pattern did not fully match experimental activity. Although A1 and A2 remained inactive, compounds A3–A6 showed MIC values of 7.96, 3.99, 2.82, and 1.65 μM, respectively, and were therefore experimentally active. This discrepancy indicates that, in the *S. agalactiae* task, the combination of a high prospective threshold and low similarity to the training set resulted in overly conservative decisions that failed to prioritize several active external compounds. Notably, several external compounds shared identical calibrated probabilities despite different raw probabilities, reflecting the piecewise-constant mapping introduced by isotonic regression on a limited calibration set. For transparency, both *p*_raw_ and *p*_active_ are therefore reported in Table 2.

### 2.4. Threshold Diagnostics for S. agalactiae Task

**Figure 4:**
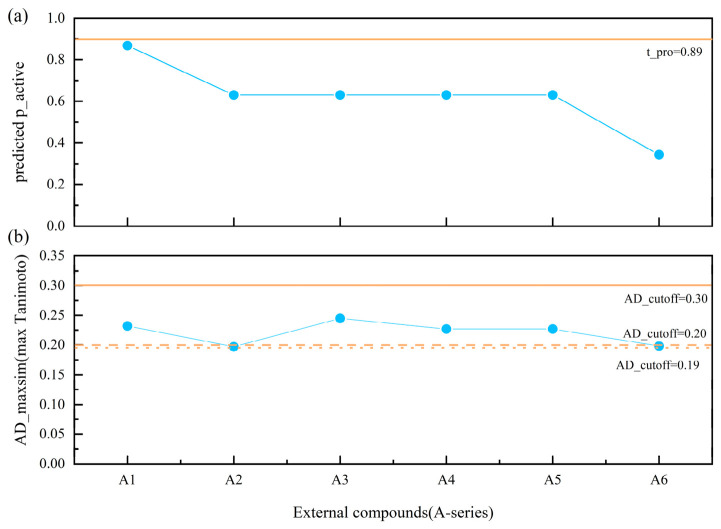
Threshold diagnostics for the external *Streptococcus agalactiae* compounds (A1–A6): (**a**) calibrated activity probabilities (*p*_active_) relative to the prospective threshold *t*_pro_; The blue dots connected by the blue line indicate the predicted pactive values for individual compounds, and the orange horizontal line indicates the prospective threshold (*t*_pro_ = 0.89). (**b**) applicability-domain similarities (AD_maxsim_) relative to the primary and relaxed AD cutoffs. The blue dots connected by the blue line indicate ADmaxsim values for individual compounds, and the horizontal reference lines indicate ADcutoff values of 0.30, 0.20, and 0.19.

To clarify why all six external compounds were classified as No-Go for *S. agalactiae*, the calibrated probabilities and AD values were examined jointly (Figure 4). The external compounds formed three probability levels: A1 showed the highest calibrated probability (0.8679), A2–A5 were mapped to 0.6300, and A6 had a lower value of 0.3429. Nevertheless, none of these values reached the predefined prospective threshold (*t*_pro_ = 0.89), meaning that all compounds failed at the probability-decision level even before AD constraints were fully considered.

The AD analysis further showed that the external compounds were only weakly similar to the *S. agalactiae* training set, with AD_maxsim_ values between 0.1967 and 0.2448. Under the primary threshold of 0.30, all compounds remained outside the applicability domain. When the AD cutoff was relaxed to 0.20, A1 and A3–A5 could be considered in-domain, whereas A2 and A6 still remained outside. When the threshold was further relaxed to 0.19, all six compounds became in-domain. Even under these relaxed settings, however, none of the compounds met the calibrated probability threshold required for a Go decision. These analyses indicate that, for this external series, failure to reach the high prospective probability threshold was the primary determinant of the uniformly conservative No-Go outcome, while AD served as an additional indicator of extrapolation risk rather than the sole cause of rejection.

### 2.5. External Decision-Error Summary

When viewed at the level of final decision behavior, the external closed-loop validation revealed two contrasting but informative decision patterns (Table 3). For *S. aureus*, the workflow was permissive: five compounds were classified as Go, but only one of them, A6, was experimentally active. Thus, the primary decision rule produced one true Go decision, four false Go decisions, one true No-Go decision, and no false No-Go decisions. In contrast, for *S. agalactiae*, the workflow was conservative: all six compounds were classified as No-Go, although four compounds, A3–A6, were experimentally active. Therefore, the primary decision rule produced two true No-Go decisions and four false No-Go decisions, with no true Go or false Go decisions.

Taken together, these results show that the practical behavior of the decision-oriented workflow depended strongly on both dataset context and extrapolation regime. For the larger *S. aureus* dataset, the workflow preserved some prioritization utility, but it did not reliably separate active from inactive compounds within the external tylosin-derivative series. For the smaller *S. agalactiae* dataset, the workflow primarily functioned as a conservative risk filter, but at the cost of missing several experimentally active compounds. These organism-specific differences support interpreting the workflow as a transparent decision-support framework with bounded transferability rather than as a universally reliable prospective screening solution.

### 2.6. Sensitivity of Go/No-Go Decisions to Probability and AD Cutoffs

To further examine whether the external decision outcomes were driven mainly by the probability threshold, the applicability-domain cutoff, or their combination, an exploratory sensitivity analysis was performed using alternative probability and AD settings (Table S4). For *S. aureus*, all six external compounds were already within the primary applicability domain; therefore, relaxing the AD cutoff from 0.30 to 0.20 or 0.19 did not change the decision outcomes. Lowering the probability threshold from *t*_pro_ to *t*_internal_ classified all six compounds as Go, but this increased false Go decisions without recovering additional active compounds. This indicates that the main limitation of the *S. aureus* workflow was optimistic probability-based prioritization rather than insufficient AD coverage.

For *S. agalactiae,* the primary rule classified all compounds as No-Go because none of the external compounds satisfied both the high probability threshold and the primary AD cutoff. Replacing *t*_pro_ with the lower *t*_internal_ alone did not change the outcome when AD_cutoff_ = 0.30, because all compounds remained outside the primary applicability domain. When the AD cutoff was relaxed, several active compounds were recovered as Go candidates, but false Go decisions also appeared. These results indicate that the conservative behavior of the *S. agalactiae* workflow resulted from the combined effects of low training-set similarity and a stringent probability threshold. Overall, the sensitivity analysis supports the interpretation that the primary Go/No-Go rule represents a transparent but threshold-dependent resource-allocation strategy rather than a universally optimal screening rule.

## 3. Discussion

### 3.1. Decision-Oriented Closed-Loop Screening Framework

The main contribution of this study is the prospective evaluation of a transparent, decision-oriented screening workflow for antibacterial prioritization [19,20]. The workflow integrates several established components, including Morgan fingerprint-based molecular representation, organism-specific MLP classification, probability calibration, applicability-domain assessment, explicit Go/No-Go decision rules, and experimental MIC validation [21]. By linking these components into a closed-loop framework, the study moves beyond retrospective model evaluation and examines how model outputs behave when translated into practical screening decisions under limited experimental-resource settings [15,19,22,23].

This distinction is important because internal discrimination metrics alone do not determine whether a model can support experimental allocation in prospective antibacterial discovery. In medicinal and veterinary chemistry practice, researchers often need to prioritize a small number of analogs within a focused scaffold series rather than screen a randomly sampled chemical collection [21,24,25]. In the present workflow, calibrated probabilities were not used merely as ranking scores, but were converted into explicit Go/No-Go decisions together with applicability-domain constraints. The subsequent MIC validation of the A1–A6 tylosin-derivative series therefore provided a direct test of decision behavior rather than only a test of retrospective ranking performance [20,26].

The external validation results showed that the workflow was informative but not uniformly reliable as a prospective activity predictor. For *S. aureus*, the model included the only experimentally active compound among the Go candidates, but also produced several false Go decisions. For *S. agalactiae*, the primary rule was overly conservative and missed several active compounds. These contrasting outcomes indicate that the value of the workflow lies in making model-guided prioritization transparent, testable, and diagnostically interpretable [21,23]. Thus, the present study should be viewed as a closed-loop decision-support and model-limitation analysis rather than as evidence that the workflow is already a high-confidence stand-alone screening tool [18,27].

### 3.2. Organism-Specific Decision Behaviors

A central finding of this study is that the same decision-oriented workflow produced distinct external decision behaviors for the two organisms. For *S. aureus*, the model was trained on a substantially larger dataset and showed stronger and more stable internal out-of-fold performance. Under external validation, the workflow retained some practical value by assigning A6, the only experimentally active compound in this external series, to the Go category. However, four additional compounds were also classified as Go despite being experimentally inactive. This indicates that, although the model preserved prioritization value to some extent, the probability-based decision rule was not sufficiently selective to distinguish active from inactive analogs within this focused tylosin-derivative series [21,23].

In contrast, the *S. agalactiae* task presented a different type of limitation. Although the internal validation metrics suggested that the task was learnable, the dataset was much smaller and the external analogs showed low similarity to the training distribution. Under the primary rule, none of the six tylosin derivatives reached the combined probability and applicability-domain requirements for a Go decision. However, MIC testing showed that A3–A6 were experimentally active. Thus, the workflow did not generate false Go decisions for *S. agalactiae*, but it produced four false No-Go decisions, indicating conservative under-selection in a low-data extrapolation regime [18,25].

These contrasting outcomes illustrate that internal model quality does not translate uniformly into prospective decision reliability. Even when the same molecular representation, model architecture, calibration strategy, and Go/No-Go rule are used, external decision behavior can differ substantially depending on dataset size, training-set coverage, and the relationship between the external compounds and the learned chemical space [23,24]. In practical screening terms, the *S. aureus* model behaved more permissively, increasing the risk of false prioritization, whereas the *S. agalactiae* model behaved more conservatively, increasing the risk of missing active candidates. Therefore, the usefulness of such a workflow should be judged not only by internal ROC-AUC or PR-AUC, but also by its external decision-error profile under realistic prioritization conditions [21,25].

### 3.3. Calibration, Applicability Domain and Extrapolation Risk

Probability calibration was introduced in this study to improve the interpretability of model outputs and to support threshold-based decision-making. Under internal validation, calibration improved Brier score and expected calibration error for both organism-specific tasks, indicating that the calibrated probabilities were more consistent with observed outcome frequencies. This is important because a probability-driven decision rule requires outputs that are more than just rank scores; they must retain at least some approximate meaning as confidence estimates [21]. Similar concerns about confidence reliability in molecular prediction have also been emphasized in recent uncertainty-aware and benchmarking studies [21,24,26].

However, the external validation results also make clear that calibrated probability remains conditional on the data distribution under which it is learned. In the *S. aureus* task, compounds with high calibrated probabilities were not always experimentally active, suggesting that calibration improved the interpretability of the model within the internal setting but did not fully prevent optimistic confidence when the model was transferred to a new analog series [21,28]. In the *S. agalactiae* task, the opposite pattern was observed: the calibrated probabilities were conservative and failed to prioritize several active compounds. Together, these two outcomes indicate that calibration alone cannot guarantee threshold stability when prospective compounds differ from the effective training distribution [16,23]. The threshold sensitivity analysis further supported this interpretation by showing that lowering the probability threshold could change external resource-allocation behavior, but did not uniformly improve decision reliability across the two organisms.

This point is particularly relevant in antibacterial discovery, where public bioactivity data are often heterogeneous with respect to strains, assay conditions, and chemical series [19]. Under such circumstances, a calibrated probability should not be interpreted as a universally portable estimate of success. Instead, it should be treated as a decision-support quantity whose usefulness depends on whether the external compounds are sufficiently represented by the structure–activity patterns learned from the training data [21,24]. The present results therefore support the view that calibration is necessary for probability-based prioritization, but not sufficient for robust extrapolative decision-making [15,17,23].

The similarity-based applicability-domain analysis added an important layer of interpretability to the workflow. In the present study, AD did not simply operate as a binary acceptance filter; instead, it helped indicate whether a compound was close enough to the training distribution for model outputs to be interpreted with greater confidence [29]. This distinction is important. In many practical workflows, AD is sometimes treated as if it can directly determine whether a prediction is valid or invalid. The present results suggest a more nuanced role, which is consistent with both classic QSAR practice and more recent AD discussions in molecular AI [27,29,30]. Thus, AD_maxsim_ was interpreted as an extrapolation-risk indicator rather than as an absolute criterion for determining whether a compound should be active or inactive.

For *S. aureus*, the external compounds were generally well within the primary applicability domain, yet several high-probability Go compounds still failed experimentally. This indicates that being inside the domain does not guarantee predictive success, especially when the external evaluation involves subtle activity differences within a focused analog series [24,29]. For *S. agalactiae*, by contrast, the low AD similarities correctly signaled that the external compounds were far from the effective training space. In this case, AD helped identify that the resulting No-Go decisions were being made under conditions of limited extrapolative support [23]. Even when the AD threshold was relaxed, however, the external compounds still failed to meet the probability criterion, confirming that AD was informative but not solely responsible for the final conservative outcome. The sensitivity analysis showed that relaxing the AD cutoff could recover several active *S. agalactiae* compounds under the lower internal probability threshold, but this also introduced false Go decisions and still failed to recover A6 because of its low calibrated probability.

These findings suggest that AD should be interpreted primarily as a marker of extrapolation risk. Its value lies in constraining how strongly model probabilities should be trusted, rather than in replacing the probability model itself [29,30]. For practical antibacterial prioritization, AD can therefore serve as a transparency tool, it helps distinguish between compounds that are rejected because they appear weak within a known region of chemical space and compounds that are rejected because the model is being asked to extrapolate beyond its reliable experience [23,27].

### 3.4. Resource Allocation, Limitations and Future Directions

One of the most practically important implications of this work is that the utility of a predictive screening workflow should be assessed in terms of how it changes experimental allocation rather than solely in terms of retrospective model metrics. In the current study, the closed-loop design exposed two different allocation behaviors. In *S. aureus*, the workflow would have directed experimental effort toward several compounds, including one truly active analog, but at the cost of testing multiple false-priority compounds. In *S. agalactiae*, the workflow would have protected resources by issuing a uniformly conservative decision, but at the cost of missing several genuinely active compounds. This kind of trade-off is highly relevant to antibacterial discovery, where experimental budgets are limited and prioritization is often the central practical objective [19,21,22].

This contrast highlights that the practical objective of model-guided screening is not simply maximizing internal AUC, but managing the trade-off between false prioritization and false rejection under realistic resource constraints [21]. The external decision-error summary and threshold sensitivity analysis further show that this trade-off was organism-specific: the *S. aureus* workflow mainly increased false prioritization, whereas the *S. agalactiae* workflow mainly increased false rejection. In some projects, a permissive strategy may be acceptable if the goal is to preserve sensitivity and identify at least a subset of active compounds. In other settings, especially when testing capacity is extremely limited, a conservative strategy may be preferable, even if it increases the risk of missed actives. The current workflow provides a concrete example of how calibrated thresholds and AD criteria can be used to make these trade-offs explicit [15,23,29].

Accordingly, the most appropriate interpretation of the present workflow is not that it serves as a high-confidence activity predictor in all settings, but that it acts as a structured decision-support framework for prioritization under uncertainty. This is particularly relevant in veterinary antibacterial discovery, where experimental throughput is often limited and compound series are frequently narrow and scaffold-focused, while resistance-aware treatment decisions for mastitis-associated pathogens are increasingly emphasized [31,32,33,34]. In that sense, the workflow is most useful when treated as a formalized allocation mechanism rather than as a promise of uniformly high external hit rates [26,28].

Several limitations of this study should be acknowledged. First, the external validation set was small and consisted of only six tylosin derivatives. Although this focused design was useful for analog-level prospective testing, it limits the statistical generalizability of the observed decision patterns. Second, the detailed chemical structures and synthetic routes of A1–A6 are currently associated with ongoing intellectual-property protection and therefore are not disclosed in the present manuscript. This limits structure-level reproducibility and prevents a full structure–activity relationship analysis of the external analog series, although the coded compounds were used only for prospective validation and were not involved in model training, calibration, threshold selection, or applicability-domain definition. Third, the public MIC datasets used for model development likely contain heterogeneity in strains, assay conditions, and reporting conventions, which may reduce the portability of learned probability thresholds across different external series [21]. In addition, the MIC ≤ 10 μM threshold should be interpreted as an operational early-discovery cutoff rather than a clinical susceptibility breakpoint. Fourth, the present workflow was constructed at the organism level and did not include resistance-phenotype-specific modeling, such as MRSA-specific prediction, because strain-level resistance annotations were not consistently available in the public MIC records. Finally, the predictive framework relied on Morgan fingerprints and a relatively simple MLP architecture. While this design was suitable for building a transparent workflow, it does not exclude the possibility that other representation-learning approaches or ensemble strategies could yield more robust performance [20,24,26].

Future work should therefore focus on iterative closed-loop refinement rather than one-time model deployment. A particularly important next step would be to feed experimentally validated tylosin-series data back into the training set, retrain the models, and re-estimate prospective thresholds under a chemical space that better reflects the intended screening domain [21]. Additional external validation on larger and more diverse tylosin-derived analog series would help determine whether the contrasting optimistic and conservative behaviors observed here are organism-specific, dataset-specific, or more broadly characteristic of low-data antibacterial extrapolation [18,23]. After intellectual-property protection is completed, disclosure of structural information would also enable more detailed structure–activity relationship analysis and structure-level reproducibility assessment of the external analog series. Beyond this, future studies should construct resistance-phenotype-labeled datasets, including MRSA-specific *S. aureus* subsets when sufficient MIC and resistance annotations are available, to evaluate whether model-guided prioritization differs between susceptible and drug-resistant strains. Future work may also benefit from comparing multiple baseline and graph-based models, integrating richer uncertainty quantification methods, and exploring task-specific threshold selection strategies that explicitly optimize prospective allocation under different experimental budgets [16,17,20,28].

Taken together, these limitations define the scope of the present workflow and highlight the need for iterative closed-loop refinement. The current results provide a practical starting point for an iterative screening paradigm in which data generation, model updating, probability recalibration, and threshold redefinition are linked in a continuous closed loop [15,19]. From a methodological perspective, that direction is also aligned with broader recommendations in molecular ML that stress prospective validation, uncertainty-aware decision support, and evaluation under realistic distribution shift rather than overreliance on convenient internal splits [18,27].

## 4. Materials and Methods

### 4.1. Data Sources and Curation

Publicly available minimum inhibitory concentration (MIC) records for *Staphylococcus aureus* and *Streptococcus agalactiae* were collected and integrated to construct organism-specific datasets for model development and internal validation. The primary data sources included ChEMBL (European Molecular Biology Laboratory’s European Bioinformatics Institute, Hinxton, UK; https://www.ebi.ac.uk/chembl/; accessed on 20 March 2026) and PubChem BioAssay (National Center for Biotechnology Information, National Library of Medicine, National Institutes of Health, Bethesda, MD, USA; https://pubchem.ncbi.nlm.nih.gov/; accessed on 20 March 2026), which contain compound-level chemical structures and antibacterial bioactivity annotations. Records were retrieved using organism-specific activity queries combining the target organism name with MIC-related antibacterial activity terms. Only records with a clearly defined test organism, a valid chemical structure, and a convertible MIC value were retained.

To reduce bias introduced by inconsistent chemical representations, all molecular structures were standardized before modeling. SMILES strings were canonicalized, and salts, mixtures, inorganic fragments, duplicate structural representations, and entries with ambiguous or unresolvable chemical structures were removed when they could not be reliably converted into a single valid compound representation. Records lacking usable SMILES information or failing structure parsing were excluded.

MIC values reported in different units were harmonized into μM whenever the corresponding molecular structure and molecular weight allowed reliable unit conversion. Records with missing MIC values, non-convertible units, incomplete concentration information, or inconsistent activity annotations were discarded. Non-exact MIC records, including censored values reported only with “>”, “<”, “≥”, or “≤”, and range-based MIC values that could not be unambiguously converted to a single numerical value, were excluded from model training. When multiple MIC records were available for the same standardized compound against the same organism, the median MIC value was used as the compound-level representative value to reduce the influence of inter-study variability, assay heterogeneity, and extreme observations.

To enable prospective prioritization using a binary classification framework, continuous MIC values were converted into binary activity labels. Compounds with MIC ≤ 10 μM were labeled as active, whereas compounds with MIC > 10 μM were labeled as inactive. This MIC ≤ 10 μM cutoff was used as an operational early-discovery activity threshold rather than a clinical susceptibility breakpoint. The threshold was selected to identify compounds with sufficient in vitro potency for prioritization while allowing heterogeneous public MIC records to be converted into a consistent binary classification task. The key characteristics of the curated datasets, including sample size, positive rate, classification threshold, and organism-specific decision thresholds, are summarized in Table 1.

### 4.2. Molecular Representation and Classification Modeling

Molecules were represented using Morgan fingerprints, equivalent to extended-connectivity fingerprints with radius 3 (ECFP6), calculated from canonical SMILES strings with a fingerprint length of 2048 bits. The same fingerprint-generation procedure was applied to both the public training compounds and the external tylosin-derived analogs. For the coded external analogs A1–A6, fingerprints were generated from internally available standardized structures for prospective prediction and applicability-domain analysis; however, the detailed chemical structures are not disclosed in the present manuscript because of ongoing intellectual-property protection. The coded analogs were not used in model training, calibration, threshold selection, or applicability-domain cutoff definition.

Separate binary classification models were developed for *S. aureus* and *S. agalactiae*. The predictive model was implemented as a multilayer perceptron (MLP) with an input dimension of 2048, two hidden layers of 512 neurons, and one sigmoid output node. Hidden layers used rectified linear unit (ReLU) activation, and dropout regularization was applied with a dropout rate of 0.2 to reduce overfitting. The output layer generated the raw predicted probability of activity, denoted as *p*_raw_.

Internal model performance was assessed using five-fold stratified cross-validation in an out-of-fold (OOF) framework. In each fold, the model was trained on the training subset and then used to predict the held-out validation subset. OOF predictions from all folds were combined to estimate internal discrimination, calibration, and early-enrichment performance under a setting that more closely reflects prediction on unseen compounds. The external A1–A6 analogs were kept completely separate from this internal validation procedure and were used only after model development was completed.

All computational analyses were performed in a conda environment (gbsdl) using Python 3.11. Molecular standardization and Morgan fingerprint generation were conducted using RDKit (version 2024.03). Data processing and numerical analyses were performed using pandas (version 2.3) and NumPy (version 2.3). Model construction, cross-validation, probability calibration, threshold selection, and performance evaluation were performed using scikit-learn (version 1.7) and PyTorch (version 2.8). PyTorch Lightning (version 2.5) was used to support neural-network model training. Figures were generated using Matplotlib (version 3.10).

### 4.3. Probability Calibration and Decision Thresholds

Because the intended use of the model was prospective compound prioritization rather than retrospective ranking alone, probability calibration was performed to improve the interpretability of model outputs. Calibration was implemented within a nested procedure to avoid information leakage. Specifically, within each outer training fold, an internal stratified cross-validation process was used to generate out-of-fold raw probabilities and corresponding labels for calibration-model fitting. The fitted calibration model was then applied only to convert predictions for the outer validation fold.

Isotonic regression was used as the default calibration method, with out-of-bounds values clipped to the nearest fitted interval. When isotonic regression was unstable because of limited data size or insufficient probability diversity, Platt scaling was used instead. The resulting calibrated probability of activity was denoted as *p*_active_, whereas the uncalibrated model output was denoted as *p*_raw_.

Model discrimination was quantified using the area under the receiver operating characteristic curve (ROC-AUC) and average precision (AP). Calibration performance was further assessed using the Brier score, expected calibration error (ECE), and reliability diagrams based on out-of-fold predictions (Table S1 and Figure S1). To convert calibrated probabilities into actionable screening decisions, two organism-specific thresholds were defined before external validation. The first threshold, *t*_internal_, was selected from out-of-fold calibrated probabilities by maximizing the F1 score and was used as an internal reference threshold. The second threshold, *t*_pro_, was selected to prioritize precision in prospective screening and was defined as the minimum calibrated probability required to achieve a target precision of 0.90 on the out-of-fold predictions.

The target precision of 0.90 was chosen to represent a conservative prioritization mode in which false Go decisions are minimized when experimental follow-up capacity is limited. This setting was not intended to represent a universally optimal threshold for all early-discovery scenarios. In contexts where avoiding false rejection is more important than reducing false prioritization, a less stringent threshold may be preferable. Therefore, threshold sensitivity analyses were conducted to evaluate how alternative probability thresholds and applicability-domain cutoffs affected external Go/No-Go outcomes. The organism-specific thresholds used in the primary decision rule are reported in Table 1.

### 4.4. External Prospective Validation and MIC Determination

To evaluate the practical transferability of the model in a realistic compound-prioritization setting, six tylosin-derived analogs designed from the tylosin scaffold were selected as an external prospective validation set and coded as A1–A6. These compounds were not involved in public-data curation, model training, internal performance evaluation, probability calibration, threshold selection, or applicability-domain cutoff definition. Because the complete chemical structures and synthetic routes of A1–A6 are associated with an ongoing intellectual-property protection process, they are not disclosed in the present manuscript. Internally retained standardized molecular structures were used only for molecular fingerprint generation, prospective prediction, and applicability-domain analysis.

Experimental MIC validation was performed against *Staphylococcus aureus* and *Streptococcus agalactiae* using a broth microdilution procedure guided by Clinical and Laboratory Standards Institute (CLSI) recommendations. The tested strains were *S. aureus* ATCC 29213 and *S. agalactiae* ATCC 13813, both obtained from the China Medical Microbial Culture Collection and Management Center. Frozen *S. aureus* cultures were revived in Mueller–Hinton (MH) broth, whereas *S. agalactiae* was revived in MH broth supplemented with 5% serum. The two strains were subsequently streaked onto MH agar and MH agar supplemented with 5% sheep blood, respectively, and incubated at 37 °C. After three to five consecutive passages, bacterial suspensions were prepared and diluted to approximately 5 × 10^5^ to 1 × 10^6^ CFU/mL for subsequent inoculation.

The test compounds were first dissolved in dimethyl sulfoxide (DMSO; Aladdin, Shanghai, China) to prepare stock solutions and were then subjected to two-fold serial dilution to 256 μg/mL for subsequent use. Gradient dilutions were performed in 96-well plates, with final test concentrations ranging from 128 to 0.0625 μg/mL. The final solvent concentration in the assay wells was maintained at 2 μL/mL, and the same solvent concentration was used in the solvent-control wells. Each 96-well plate included a growth control without test compound, a sterility control without bacterial inoculum, a solvent control, and a tylosin control. After inoculation, the plates were incubated at 37 °C for 18–24 h. MIC was defined as the lowest compound concentration that completely inhibited visible bacterial growth. All MIC assays were performed in three independent biological replicates, with three technical replicates in each experiment. The final MIC value was calculated as the median of all replicate measurements and converted to μM for consistency with the model-training data. The same activity criterion used during model development was applied in the external validation, with MIC ≤ 10 μM defined as active.

### 4.5. Applicability Domain and Go/No-Go Rules

To characterize extrapolation risk for new compounds, a similarity-based applicability domain (AD) analysis was incorporated into the prospective screening workflow. For each external compound, the Tanimoto similarity between its Morgan fingerprint and the fingerprints of all compounds in the corresponding organism-specific training set was calculated. The maximum similarity value was defined as AD_maxsim_.

(1) $${AD}_{maxsim}(x)=\underset{x_{i}\in \mathrm{Train}}{\max}Tanimoto(x,x_{i})$$

where x represents an external compound and x_i_ represents a compound in the organism-specific training set.

In the primary analysis, compounds were considered to have sufficient structural support from the training distribution when AD_maxsim_ ≥ 0.30. This cutoff was used as an empirical similarity threshold to flag compounds with limited training-set support and was not intended to define an absolute boundary of prediction validity. Because applicability-domain criteria can affect external prioritization decisions, additional relaxed cutoffs of 0.20 and 0.19 were examined in sensitivity analyses, particularly for the *S. agalactiae* external compounds, which showed generally low similarity to the training data.

Prospective decisions were defined using a joint probability–domain rule. A compound was classified as Go only when both of the following conditions were satisfied: *p*_active_ ≥ *t*_pro_ and AD_maxsim_ ≥ AD_cutoff_. Otherwise, the compound was classified as No-Go. Therefore, a No-Go outcome could result from a low calibrated activity probability, insufficient applicability-domain support, or both. The values of *p*_raw_, *p*_active_, AD_maxsim_, Go/No-Go decision, and experimentally measured MIC for all external compounds are summarized in Table 2.

### 4.6. Threshold and Applicability-Domain Sensitivity Analysis

Because prospective Go/No-Go outcomes may depend on the selected probability threshold and applicability-domain cutoff, an exploratory sensitivity analysis was performed to examine the robustness of the external decisions. The primary decision rule used the precision-oriented threshold *t*_pro_ together with the empirical applicability-domain cutoff of AD_maxsim_ ≥ 0.30. To evaluate the influence of these choices, alternative decision settings were examined by replacing *p*_pro_ with the internal reference threshold *t*_internal_ and by relaxing the applicability-domain cutoff from 0.30 to 0.20 and 0.19.

For each threshold combination, the number of compounds classified as Go, the number of experimentally active compounds among the Go candidates, and the number of experimentally active compounds missed as No-Go were summarized. This analysis was not intended to retrospectively optimize the decision rule after observing the external MIC results. Instead, it was used to diagnose how sensitive the prospective prioritization behavior was to the predefined probability and applicability-domain criteria. The detailed sensitivity results are provided in Table S4.

### 4.7. Early Enrichment and Scaffold-Based Evaluation

To assess the practical value of the models in prioritizing a small number of candidates for follow-up testing, early enrichment metrics were calculated from out-of-fold calibrated probabilities, including enrichment factors at the top 1% and top 5% (EF1% and EF5%) as well as Top-(k) precision. These analyses were intended to simulate limited-resource screening scenarios in which only a small fraction of ranked candidates can be experimentally evaluated. Early enrichment was interpreted as an internal prioritization metric rather than as direct evidence of external prospective reliability.

Because random or stratified splits may overestimate generalization performance in molecular prediction tasks when structurally similar compounds appear in both training and validation subsets, a stricter scaffold-based evaluation was also conducted using a Bemis–Murcko scaffold split. Under this setting, model discrimination, calibration, and early enrichment were re-evaluated on scaffold-separated test sets. The corresponding results are summarized in Table S3 and were used to assess whether calibrated probability outputs retained partial utility under a more challenging structural-extrapolation setting. This scaffold-based analysis was interpreted together with, but not as a substitute for, the prospective external MIC validation using the A1–A6 tylosin-derivative series.

## 5. Conclusions

In this study, we developed and prospectively evaluated a calibrated, decision-oriented closed-loop screening workflow for tylosin derivatives by integrating organism-specific activity prediction, probability calibration, applicability-domain analysis, explicit Go/No-Go decision rules, and experimental MIC validation against *Staphylococcus aureus* and *Streptococcus agalactiae*. Internal validation showed good discriminative performance for both pathogens, with stronger and more stable results in the larger *S. aureus* dataset. However, external validation on six synthesized tylosin derivatives revealed distinct organism-specific decision limitations. For *S. aureus*, the workflow successfully included the only experimentally active compound among the Go candidates, but also generated several false Go decisions for inactive analogs. In contrast, for *S. agalactiae*, all compounds were classified as No-Go under the primary rule despite several derivatives showing experimental activity, indicating conservative under-selection in a low-data extrapolation setting.

These findings show that calibrated probabilities and applicability-domain constraints can make model-guided antibacterial prioritization more transparent and diagnostically informative, but they do not guarantee robust external activity prediction when dataset size, chemical-space coverage, threshold transferability, and extrapolation conditions are limited. Therefore, the proposed workflow should be interpreted as an early-stage, risk-aware decision-support framework rather than as a stand-alone predictor of external antibacterial activity. Future work should incorporate newly validated tylosin-series data into iterative model updating, probability recalibration, threshold refinement, and larger prospective validation to improve the robustness of model-guided prioritization for veterinary antibacterial discovery.

## Figures and Tables

**Table 1:** Dataset characteristics, internal validation performance, and decision thresholds for the organism-specific screening models.

Organism	n	Positive Rate	ROC-AUC	PR-AUC	*t*_internal_	*t*_pro_	Target Precision	Active Threshold (μΜ)
*S. aureus*	43,889	0.443	0.907	0.882	0.42	0.85	0.9	10
*S. agalactiae*	559	0.639	0.834	0.86	0.51	0.89	0.9	10

**Table 2:** Prospective external validation results for tylosin derivatives (A1–A6), including predicted probabilities, applicability-domain similarity, Go/No-Go decisions, and experimental MIC values.

Organism	Compound	*p*_raw_	*p*_active_	AD_maxsim_	Go/No-Go at AD_cutoff_ = 0.30	MIC (μM)
*S. aureus*	A1	0.937525	0.691932	0.86	No-Go	65.63
*S. aureus*	A2	0.999868	0.891967	0.919	Go	38.51
*S. aureus*	A3	0.999987	0.920482	0.891	Go	63.67
*S. aureus*	A4	0.999989	0.920482	0.813	Go	15.98
*S. aureus*	A5	0.999989	0.920482	0.813	Go	31.96
*S. aureus*	A6	1.000000	0.968365	0.785	Go	4.67
*S. agalactiae*	A1	0.999192	0.867925	0.232	No-Go	16.41
*S. agalactiae*	A2	0.967767	0.63	0.197	No-Go	13.61
*S. agalactiae*	A3	0.9872	0.63	0.245	No-Go	7.96
*S. agalactiae*	A4	0.756906	0.63	0.227	No-Go	3.99
*S. agalactiae*	A5	0.756906	0.63	0.227	No-Go	2.82
*S. agalactiae*	A6	0.027238	0.342857	0.198	No-Go	1.65

**Table 3:** External decision-error summary of the primary Go/No-Go workflow.

Organism	True Go	False Go	True No-Go	False No-Go	Main Decision Pattern
*S. aureus*	1	4	1	0	Permissive/optimistic
*S. agalactiae*	0	0	2	4	Conservative/under-selective

True Go indicates that a compound was classified as Go and was experimentally active. False Go indicates that a compound was classified as Go but was experimentally inactive. True No-Go indicates that a compound was classified as No-Go and was experimentally inactive. False No-Go indicates that a compound was classified as No-Go but was experimentally active. Experimental activity was defined as MIC ≤ 10 μM.

## Data Availability

The curated datasets, molecular representations, data splits, prediction outputs, and external validation results are available in the Supplementary Materials. The source code and processed data can be made available from the corresponding author upon reasonable request.

## Supplementary Materials

The following supporting information can be downloaded at: https://www.mdpi.com/article/10.3390/antibiotics15070666/s1, Figure S1. Reliability diagrams for probability calibration in the OOF setting. (a) *Staphylococcus aureus*. (b) *Streptococcus agalactiae*. Orange lines denote raw probabilities, and green lines denote calibrated probabilities. The blue dashed diagonal represents ideal calibration. The x-axis shows mean predicted probability, and the y-axis shows the observed fraction of positives. Table S1. Calibration performance of raw and calibrated probabilities on OOF predictions. Table S2. (A) Early enrichment factors based on OOF predictions. (B) Top-*k* precision based on OOF predictions. Table S3. Scaffold-split evaluation of discrimination, calibration, and early enrichment. Table S4. Exploratory sensitivity analysis of external Go/No-Go outcomes under alternative probability thresholds and applicability-domain cutoffs.
