# Supplementary material for: A Decision-Oriented Calibrated Screening Workflow for Tylosin Derivatives: Closed-Loop MIC Validation Against Staphylococcus aureus and Streptococcus agalactiae
Source: Antibiotics (Basel). 2026 Jul 8;15(7):666. doi: 10.3390/antibiotics15070666 (PMC13405834; doi:10.3390/antibiotics15070666)
Supplement: Supplementary file 1 [file antibiotics-15-00666-s001.zip › antibiotics-4389164-supplementary.pdf]

Supplementary Materials:

Supplementary Note S1. Probability calibration

To obtain interpretable probabilities suitable for threshold-based decision-making, post hoc calibration was applied to the raw model outputs, and calibration performance was quantitatively compared on out-of-fold (OOF) predictions before and after calibration. For both organism-specific tasks, calibration reduced the Brier score and the expected calibration error (ECE) (Table S1). In the *Staphylococcus aureus* task, the calibration curve moved closer to the ideal diagonal after calibration, indicating improved agreement between predicted probabilities and observed positive rates across the probability range (Figure S1a). In the *Streptococcus agalactiae* task, calibration also improved overall agreement with the ideal calibration line, although some fluctuations remained because of the smaller dataset size and the limited number of samples contributing to several bins (Figure S1b). Taken together, these results indicate that calibration improved the consistency between model probabilities and empirical outcome frequencies, thereby supporting the use of the prospective threshold t_{pro} in the main screening workflow.

(a)

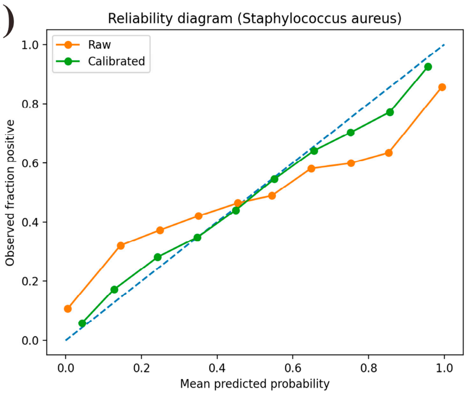

(b)

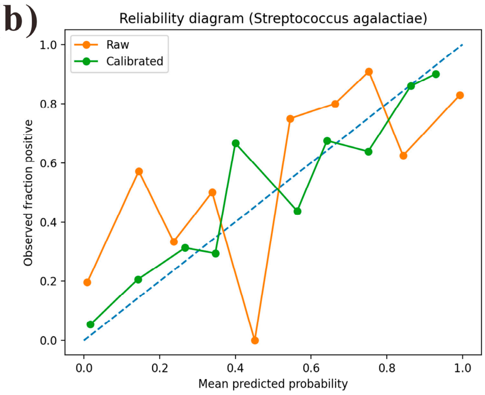

Figure S1. Reliability diagrams for probability calibration in the OOF setting. (a) *Staphylococcus aureus*. (b) *Streptococcus agalactiae*. Orange lines denote raw probabilities, and green lines denote calibrated probabilities. The blue dashed diagonal represents ideal calibration. The x-axis shows mean predicted probability, and the y-axis shows the observed fraction of positives.

Table S1. Calibration performance of raw and calibrated probabilities on OOF predictions.

organism	n	Positive rate	Brier score (raw)	Brier score (calibrated)	ECE (raw)	ECE (calibrated)	Number of bins
Staphylococcus aureus	43889	0.443	0.138	0.120	0.117	0.030	10
Streptococcus agalactiae	559	0.639	0.179	0.150	0.174	0.039	10

Supplementary Note S2. Early enrichment analysis

To simulate low-resource screening scenarios in which only a small fraction of ranked candidates can be experimentally validated, early enrichment metrics were calculated from OOF prediction probabilities, including enrichment factors at the top 1% and top 5% (EF1% and EF5%) as well as Top-*k* precision. For *S. aureus*, the model showed strong enrichment in the top-ranked region, with EF1% = 2.20 and EF5% = 2.19, while Top-10 and Top-50 precision were both 1.00 (Tables S2A and S2B). These results suggest that the model could substantially improve hit identification when only a small number of candidates can be prioritized for follow-up testing. For *Streptococcus agalactiae*, enrichment was also observed, although the estimates were more variable because of the smaller dataset size and the limited number of compounds included in the top-ranked fractions. Overall, these results support the practical value of the workflow for prioritizing compounds under limited experimental capacity.

Table S2A. Early enrichment factors based on OOF predictions.

Organism	Metric	Top fraction	Number in top set	Hits in top set	Precision in top set	Enrichment factor
Staphylococcus aureus	EF1%	0.01	439	427	0.973	2.198
Staphylococcus aureus	EF5%	0.05	2,194	2,123	0.968	2.186
Streptococcus agalactiae	EF1%	0.01	6	5	0.833	1.305
Streptococcus agalactiae	EF5%	0.05	28	24	0.857	1.342

Table S2B. Top-*k* precision based on OOF predictions.

Organism	Metric	k	Precision
Staphylococcus aureus	Top-10 precision	10	1.00
Staphylococcus aureus	Top-50 precision	50	1.00
Staphylococcus aureus	Top-100 precision	100	0.98
Streptococcus agalactiae	Top-10 precision	10	0.80
Streptococcus agalactiae	Top-50 precision	50	0.86
Streptococcus agalactiae	Top-100 precision	100	0.90

Supplementary Note S3. Scaffold-based generalization assessment

Because random or stratified data splits may overestimate generalization in molecular prediction tasks, a stricter evaluation was performed using a Bemis-Murcko scaffold split (Table S3). Under this setting, the *Staphylococcus aureus* model retained reasonable

discriminative ability, with ROC-AUC = 0.809 and PR-AUC = 0.815, and also showed substantial early enrichment, with EF1% = 1.99 and Top-1% precision = 0.989. These results suggest that the model retained partial transferability to compounds with unseen scaffolds. In contrast, the *Streptococcus agalactiae* model showed weaker scaffold-test performance, with ROC-AUC = 0.640 and PR-AUC = 0.674, indicating that extrapolation remained more difficult in the smaller data regime. Nevertheless, calibration still improved the agreement between predicted probabilities and empirical outcomes in both tasks, as reflected by reductions in both ECE and Brier score. These findings support the use of probability calibration together with applicability-domain analysis to manage extrapolation risk in low-data antibacterial screening settings.

Table S3. Scaffold-split evaluation of discrimination, calibration, and early enrichment.

Organism	n train	n test	Positive rate (test)	ROC-AUC	PR-AUC	Brier score (raw)	Brier score (calibrated)	ECE (raw)	ECE (calibrated)	EF1%	Top-1% precision	EF5%	Top-5% precision	Calibrator
Staphylococcus aureus	35,111	8,778	0.496	0.809	0.815	0.214	0.194	0.178	0.122	1.992	0.989	1.932	0.959	Isotonic
Streptococcus agalactiae	447	112	0.554	0.640	0.674	0.354	0.230	0.361	0.099	1.806	1.000	1.204	0.667	Isotonic

Table S4. Exploratory sensitivity analysis of external Go/No-Go outcomes under alternative probability thresholds and applicability-domain cutoffs.

Organism	Probability threshold	AD cutoff	Go compounds	Active compounds among Go	Inactive compounds among Go	Missed active compounds	Decision interpretation
S. aureus	t_{pro}=0.85	0.30	A2-A6	1	4	0	Primary rule; permissive for this external series
S. aureus	tintenal=0.42	0.30	A1-A6	1	5	0	Lower probability threshold increased false Go decisions
S. aureus	tintenal=0.42	0.20	A1-A6	1	5	0	AD relaxation had no effect because all compounds were already in-domain
S. aureus	tintenal=0.42	0.19	A1-A6	1	5	0	AD relaxation had no effect because all compounds were already in-domain

S. agalactiae	t_{pro}=0.89	0.30	None	0	0	4	Primary rule; conservative under-selection
S. agalactiae	tintenal=0.51	0.30	None	0	0	4	Lower probability threshold alone did not change decisions because all compounds remained outside AD
S. agalactiae	tintenal=0.51	0.20	A1, A3-A5	3	1	1	Relaxing AD recovered three active compounds but introduced one false Go decision
S. agalactiae	tintenal=0.51	0.19	A1-A5	3	2	1	Further AD relaxation increased false Go decisions and still missed A6

Activity was defined as MIC ≤ 10 μM.
"Active compounds among Go" refers to experimentally active compounds correctly prioritized as Go.
"Inactive compounds among Go" refers to false Go decisions.
"Missed active compounds" refers to experimentally active compounds classified as No-Go.
The analysis was intended to diagnose decision sensitivity to threshold choice, not to retrospectively optimize the decision rule after observing the external MIC results.
